# Endometrioid Paraovarian Borderline Cystic Tumor in an Infant with Proteus Syndrome

**DOI:** 10.1155/2015/392576

**Published:** 2015-10-19

**Authors:** Liliana Vasquez, Mariela Tello, Ivan Maza, Monica Oscanoa, Milagros Dueñas, Haydee Castro, Alan Latorre

**Affiliations:** ^1^Department of Oncology and Radiotherapy, Rebagliati Hospital, Lima, Peru; ^2^Department of Genetics, Rebagliati Hospital, Lima, Peru; ^3^Department of Gynecology-Oncology, Rebagliati Hospital, Lima, Peru; ^4^Department of Pathology, Rebagliati Hospital, Lima, Peru

## Abstract

Ovarian and paraovarian neoplasms are uncommon in children, mainly originating from germ cell tumors and, least frequently, epithelial tumors. There is an association between genital tract tumors and Proteus syndrome, a rare, sporadic, and progressive entity, characterized by a postnatal overgrowth in several tissues caused by a mosaic mutation in the AKT1 gene. We describe a 20-month-old asymptomatic infant with Proteus syndrome who developed an endometrioid paraovarian borderline cystic tumor. This is the youngest patient so far reported in the literature with this rare syndrome and an adnexal tumor of borderline malignancy. A total of nine patients have been described with female tract tumors and associated Proteus syndrome, which includes bilateral ovarian cystadenomas and other benign masses. A paraovarian neoplasm is extremely rare in children and could be considered a criterion for Proteus syndrome. Standardized staging and treatment of these tumors are not well established; however, most authors conclude that these neoplasms must be treated as their ovarian counterparts.

## 1. Introduction

Ovarian and paraovarian neoplasms are rare in children and represent less than 5% of solid tumors [1–3]. The most common age at presentation is the second decade of life and usually older girls are at greatest risk of malignancy [4]. Incidence of ovarian neoplasms in the pediatric age group is unknown, but it is estimated at 2.6 cases per 100,000 girls per year [2]. The most common histological subtype of pediatric ovarian cancer is derived from germ cells [5–7], followed by epithelial tumors, such as cystadenomas or adenocarcinomas. Primary malignant paraovarian epithelial tumors are even more rare, previously described in literature as cystadenocarcinomas with low malignant potential [8, 9].

Proteus syndrome (PS) is a rare and sporadic disorder that causes postnatal overgrowth of multiple tissues in a mosaic pattern [10].

Cohen Jr. and Hayden first described it in 1979 as “a new hamartomatous syndrome” [11]. Wiedemann et al. further explained the syndrome and named it Proteus syndrome [12]. Since its first description until today, there have only been approximately 200 diagnosed cases in developed countries. PS is a progressive disorder that most commonly affects the skeleton, skin, adipose, and central nervous systems, causing severe overgrowth and disfigurement, physical disability, and a decline in the patient's quality of life [13]. It is associated with a range of tumors, pulmonary complications, and a striking predisposition to deep vein thrombosis and pulmonary embolism [14]. Among such tumors are those derived from lymphatic tissue and ovarian tumors, which are also included in the diagnostic criteria for this syndrome.

We report an infant with PS, who developed an endometrioid borderline paraovarian cystic tumor. This is the tenth case reported worldwide presenting an adnexal mass associated with this rare syndrome and the first case reported in our region.

## 2. Case Report

**Figure 1:**
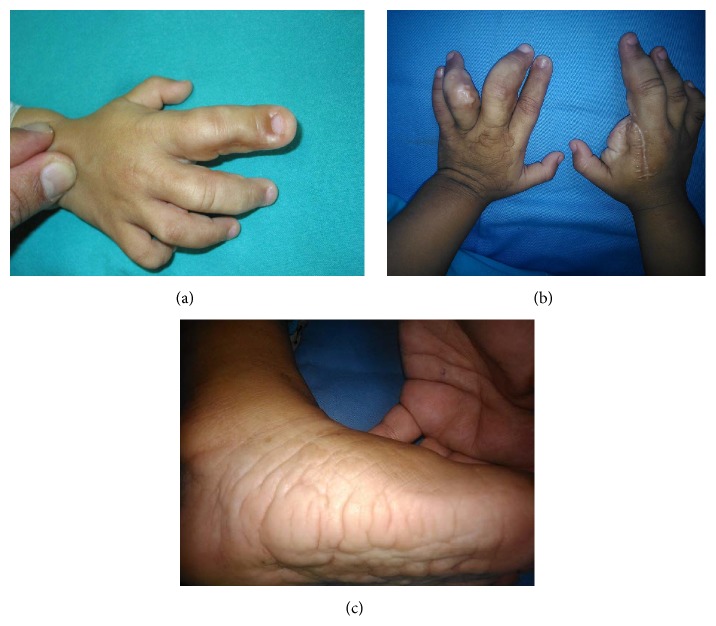
(a) Asymmetric overgrowth of the second finger of right hand. (b) After finger amputation. (c) Cerebriform connective tissue nevus of foot.

A female patient, originated from a third pregnancy and nonconsanguineous marriage, is described. Her mother was hospitalized during the second and third trimester of pregnancy, due to preterm labor and oligohydramnios. She was delivered vaginally at 36 weeks of gestation, with a birth weight of 3098 g. Upon birth, she presented normal anthropometric parameters and had no family history of any disease. At five months of age, she was noted to have abnormal augmentation of the size of her inferior limbs and asymmetric finger growth, especially involving the second finger on the right hand (Figures 1(a) and 1(b)) that required amputation at 12 months of age. Also, the patient showed several soft tissue and epidermic nevi (Figure 1(c)).

According to the clinical criteria for diagnosis of PS, our patient had the three general criteria and also had specific criteria from categories A and B (Table 1) [15, 16]. A genetic study was performed in two affected tissues, looking for the c.49G>A mutation in the AKT1 gene, but it could not been found.

**Figure 2:**
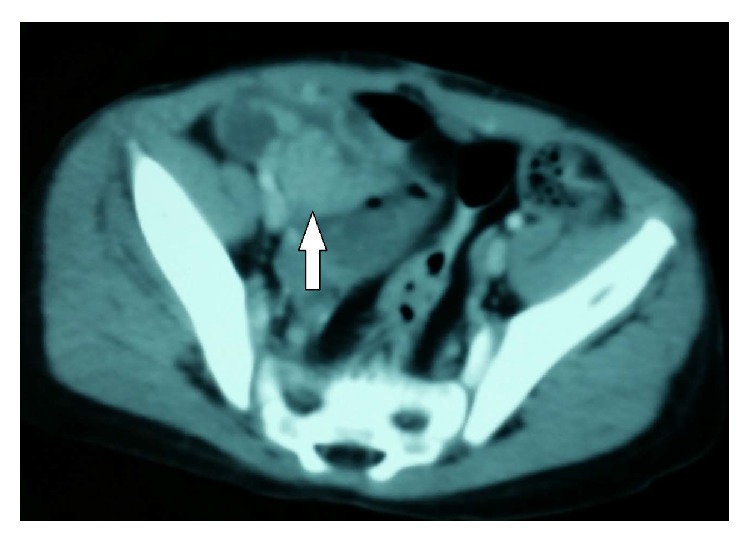
Preoperative axial contrast-enhanced CT scan showing a mass growing next to the right ovary (white arrow).

**Figure 3:**
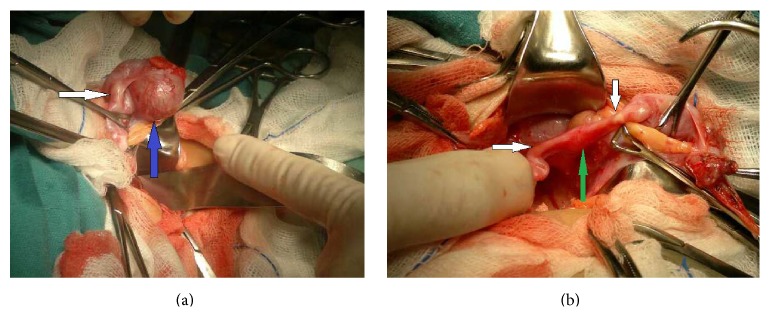
Intraoperative findings of laparotomy. (a) Paraovarian mass (blue arrow) and right tube (white arrow). Right ovary is located behind tumor. (b) After tumor excision, left and right tubes (white arrows) and normal uterus (green arrow) are shown.

**Figure 4:**
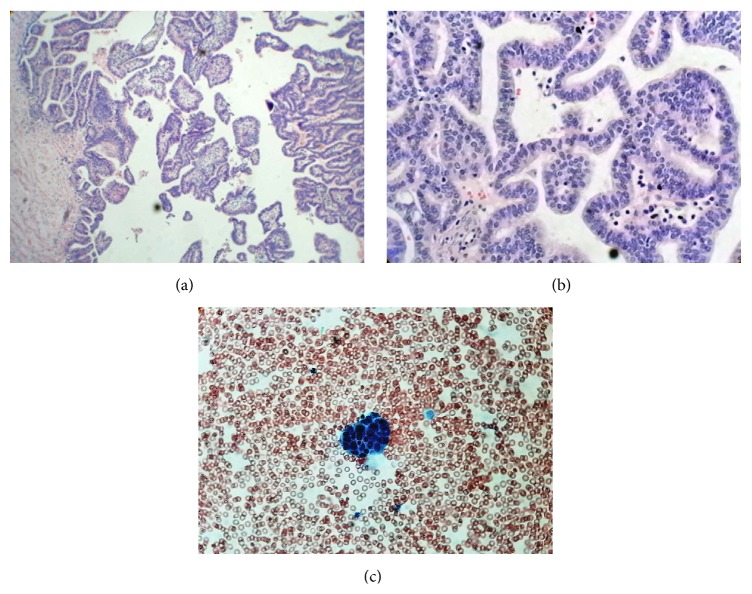
H&E stain, microscopic image. (a) 10x magnification. Architecture of paraovarian endometrioid borderline cystic tumor with villoglandular pattern. (b) 40x magnification. (c) 40x peritoneal wash. Pleomorphic, well-differentiated endometrioid groups of tumor cells, found in a peritoneal fluid.

At 20 months of age, the patient presented painless vaginal bleeding and gynecomastia; no pain or size augmentation in the abdominal region was noted. An ultrasound image showed a right solid paraovarian mass, measuring 29 × 25 × 27 mm, with scarce peripheral vascularization on Doppler imaging. The uterus and both ovaries presented no alterations. At first, CA 125 level was elevated (164 UI). A pelvic contrast-enhanced computed tomography (CT) showed a heterogeneously enhanced round mass next to the right ovary (Figure 2). No metastases were found. The patient underwent a laparotomy, where a right 2 × 2 cm violaceous paraovarian tumor was found. Excision of the tumor (tumorectomy) was completed with no evidence of rupture (Figures 3(a) and 3(b)). Omentum, peritoneal surface, and ascitic fluid samples were taken; but as fluid was scarce, peritoneal lavage was performed. Pathology was described as a well-differentiated endometrioid borderline tumor with villoglandular pattern G1 (Figures 4(a) and 4(b)); peritoneal lavage tested positive for tumoral cells, (Figure 4(c)) but no other abnormal pathological findings were made.

The patient presented a favorable postoperatory evolution and received 3 cycles of adjuvant chemotherapy administered every 21 days; a 3-hour infusion of paclitaxel 175 mg/m^2^ on days 1, 8, and 15; and carboplatin AUC 6 during day 1. At the time of this report, the patient is alive and well at 2 years of follow-up with no evidence of disease.

## 3. Discussion

Ovarian and paraovarian masses are infrequent among girls, representing less than 5% of solid tumors among children and less than 1.5% of malignant tumors [2]. Clinically, presenting symptoms are abdominal pain, a palpable tumor on a routine examination, or the presence of endocrine alterations, such as precocious puberty [17]. Diagnosis of malignant lesions generally occurs late, because of low levels of suspicion or unspecific symptoms.

According to the World Health Organization (WHO), ovarian tumors are classified into major 3 groups (surface epithelial tumors, germ cell tumors, and sex-cord stromal tumors) and other minor tumors. The most common histological group at pediatric age is the one originating from germ cells [5–7], with a great variety of histological subgroups [18]. Epithelial tumors are very rare among young girls; moreover, the finding of an endometrioid subtype, typically seen among older women [7], is even more infrequent. Also, most papers refer mainly to ovarian pathology; there is very little information regarding fallopian tubes and paratubal/paraovarian masses, this being restricted to small series and reports on clinical cases.

PS is a rare disorder, characterized by an abnormal overgrowth of several tissues, caused by a mosaic activating mutation in the AKT1 gene (c.49G>A, p.Glu17Lys) [10, 19]. Clinical diagnosis includes congenital morphological criteria, typical skin lesions, and association with tumors in postnatal life [10, 20]. This gene is in charge of sporadically activating growth of various tissues (epidermal, connective, osseous, fat, and endothelial) during embryonic development and it is characterized by partial gigantism of hands and feet, skin hemihypertrophy, epidermal nevi, subcutaneous hamartomas, macrocephaly, cranial anomalies, and connective tissue nevi. The latter is almost pathognomonic [15, 21]. Our patient met the established criteria for clinical diagnosis of PS showed in Table 1; however, the genetic testing performed could not find the target mutation in AKT1 gene.

Several benign and malignant tumors are associated with PS [10, 22], such as lipomas, haemangiomas, and lymphangiomas and, less frequently, genital tract tumors. These neoplasia cases usually occur in the second decade of life, most commonly bilateral ovarian cystadenomas or salivary gland monomorphic adenomas [15], which have an important role in establishing diagnosis for PS. Gordon et al. [23] reported a girl with PS and bilateral mucinous cystadenomas of the ovary. Furthermore, Babovic et al. [24] described the association among microdeletion of gene 10q23 and bilateral cystadenoma of the ovary and young polyposis in a teenager. Reports of patients with adnexal tumors and PS are rare and mainly describe benign tumors [25–27]. When conducting a revision of reported cases worldwide, we found only 9 cases, and one of them, reported by Raju et al. [28], belongs to a 3-year-old girl with PS, who developed a low-or-borderline malignant endometrioid cystadenoma lesion with villoglandular pattern. Development of a borderline ovarian neoplasia at a very young age supports the genetic mechanism of overgrowth seen in this syndrome and also represents the youngest patient suffering from endometrioid paraovarian cystic tumor.

Staging and treatment of epithelial paraovarian tumors among adult women are not adequately described [29] and there is even less evidence regarding the pediatric population. Its rarity in children has not allowed publishing multicentric prospective series; therefore, management is mainly based on studies involving adult patients. The majority of authors defend the fact that these tumors must be handled like their ovarian counterparts [8], that is, performing an adequate surgical staging, whether it is conservative or radical. In the present case, conservative surgery (tumorectomy) was performed due to the patient's young age, desired future fertility, and lack of unfavorable intraoperative findings, such as tumoral adherences or rupture. Surgical treatment is vital and could be curative; chemotherapy is only used in cases of patients with inoperable disease or a microscopic or macroscopic residual disease but remains controversial. Due to the presence of ascitic fluid (testing positive for tumoral cells), stage IC was established, employing adjuvant chemotherapy. Due to the fact that this disorder is rare, there is no consensus on follow-up [30], although it is accepted worldwide that this should be frequent and based on tumoral markers and pelvic imaging.

In conclusion, the finding of a paraovarian neoplasm is extremely rare in children and could be considered a clinical criterion for PS. Standardized staging and treatment of these tumors are not well established; however, most authors conclude that these neoplasms must be treated as their ovarian counterparts.

## Figures and Tables

**Table 1:** Revised Proteus syndrome diagnostic criteria (Turner et al., 2004) [15].

To make a diagnosis of PS, one must have all the general criteria and various specific criteria	
General criteria	All of the following:
	(i) Mosaic distribution of lesions
	(ii) Sporadic occurrence
	(iii) Progressive course

Specific criteria	Either
	(i) category A,
	(ii) two from category B, or (iii) three from category C

Specific criteria categories	(A) Cerebriform connective tissue nevus^a^
	(B) (1) Linear epidermal nevus
	(2) Asymmetric, disproportionate overgrowth^b^
	One or more:
	(a) Limbs:
	Arms/legs
	Hands/feet/digits
	Extremities
	(b) Hyperostoses of the skull
	(c) External auditory meatus
	(d) Megaspondylodysplasia
	(e) Viscera: spleen/thymus
	(3) Specific tumors before 2nd decade
	One of the following:
	(a) Ovarian cystadenoma
	(b) Parotid monomorphic adenoma
	(C) (1) Dysregulated adipose tissue
	Either one:
	(a) Lipomas
	(b) Regional absence of fat
	(2) Vascular malformations
	One or more:
	(a) Capillary malformation
	(b) Venous malformation
	(c) Lymphatic malformation
	(3) Lung cysts
	(4) Facial phenotype^c^
	All:
	(a) Dolichocephaly
	(b) Long face
	(c) Down slanting palpebral fissures and/or * *minor ptosis
	(d) Low nasal bridge
	(e) Wide or anteverted nares
	(f) Open mouth at rest

^a^Cerebriform connective tissue nevi are skin lesions characterized by deep grooves and gyrations as seen on the surface of the brain.

^b^Asymmetric, disproportionate overgrowth should be carefully distinguished from asymmetric, proportionate overgrowth (see Discussion for recommended methods of distinction).

^c^The facial phenotype has been found, to date, only in PS in patients who have mental deficiency and, in some cases, seizures and/or brain malformations.
